# Editorial: Interactions Between Education, Practice of Physical Activity and Psychological Well-Being

**DOI:** 10.3389/fpsyg.2020.00829

**Published:** 2020-04-28

**Authors:** Adrià Muntaner-Mas, Pere Palou, Pedro L. Almeida, Alexandre Garcia-Mas

**Affiliations:** ^1^GICAFE “Physical Activity and Exercise Sciences Research Group”, University of Balearic Islands, Balearic Islands, Spain; ^2^PROFITH “PROmoting FITness and Health Through Physical Activity” Research Group, Department of Physical Education and Sports, Faculty of Sport Sciences, University of Granada, Granada, Spain; ^3^ISPA - Instituto Universitário, Lisbon, Portugal

**Keywords:** physical activity, exercise psychology, psychological factors, cognition, physical education

Physical activity is scientifically recognized as a modifiable habit lifestyle with a wide array of benefits for a human being. Thus, there is strong evidence that performing human movement has a positive impact on the lives of people. Traditionally, these implications have been associated only on a physical dimension, but in recent times there is much evidence on mental or brain level, specifically on certain aspects of human behavior and personality traits. Additionally, the interest between this modifiable factor (physical activity) and the educational field is being the target of recent investigations. This Research Topic aims to update the scientific knowledge within this triangle built up around physical activity, psychological well-being, and education. Specifically, it yielded 16 articles, which are the result of a process of free sending, peer reviewing and modification of the manuscripts, embedded in three strands of research. Firstly, three articles focused on the role of physical activity in different population contexts. Secondly, six articles examined the effects of physical activity, sport, and exercise on psychological variables. Thirdly, seven articles examined the implications of physical activity in a variety of educational contexts. These three research strands are presented below.

Worldwide insufficient physical activity is worrisome (Guthold et al., 2018). In this scenario, the use of electronic media has been noted as an important risk factor for a sedentary lifestyle. Zagalaz-Sánchez et al. analyze the possible relation between the misuse of smartphones and the reduction in the practice of physical activity. Although they found scarce scientific evidence, it seems that improper use of this technology might be linked with lower levels of physical activity. In addition to these intriguing facts, the situation is even more alarming in the female gender, being much less physically active (Serra et al., 2018). Thus, Serra et al. focused the research on determining factors affecting interest in pursuing a degree in Physical Activity and Sport Science (PASS) among teenage students. They found a lack of interest by young women in studying a degree in PASS in comparison with boys, which was partially explained by perceived social supports and positive conceptions of outcome expectations. Likewise, physical activity could also play an important role from an early age, thereby, Palou et al. suggested that parent's reports of their children's physical activity levels were associated with relevant health markers (physical fitness and body composition indicators) in preschool children.

The second strand of our Research Topic has attempted to advance knowledge in sports psychology by investigating relationships across some psychological factors in a diverse context. Sports performance is a subsequent result of multiples factors, among them psychological variables play an important role, such as competitive anxiety or flow disposition (Swann et al., 2018; Rice et al., 2019). Thus, as far as we know, Ponseti et al. have been the first ones in demonstrating by means of a Bayesian network two probability trees that have extrinsic motivation and motivation at the top, while the anxiety/activation due to worries about the performance was found clearly at the bottom of the probabilities. In addition, Fernando Garcia et al. found a positive link between dispositional flow and performance in ironman athletes and Ribeiro Contreira et al. demonstrated that young athletes' and coaches' sport satisfaction is strongly influenced by the satisfaction of their basic psychological needs. Our Research Topic also explores some psychological variables in relation to physical activity and exercise in a non-sport population. For instance, Castro-Sánchez et al. developed an explicative model suggesting that practicing physical activity in a natural environment is a key to acquire healthy patterns and diminish the consumption of harmful substances. However, as evidenced by Chacón-Cuberos et al. the practice of physical activity seems to be not only beneficial for a physical health dimension, as well as, for its role to develop a better resilience capacity, tolerance to adversity and the positive acceptance of changes. In a further step, Aguirre-Loaiza et al. have demonstrated the effects on executive functions, and mainly, in the emotional contextual recognition with a single bout of moderate-high intensity exercise.

Within the scope of this Research Topic, the third strand was centered in an education context. A group of researchers examined theoretical approaches to understand the relation of self-concept and physical activity on students. Fernández-Bustos et al. observed that physical activity had a positive and indirect effect on self-concept and direct effects on body dissatisfaction and physical self-concept. Onetti-Onetti et al. confirmed this positive relationship between physical activity self-concept, however, they also found a negative relationship between the self-concept dimensions and the amount of time the adolescent spends sitting. These results reflect the usefulness of promoting physical activity to achieve a positive self-concept and promote psychological well-being in both adolescents and university students.

An innovative methodological approach was conducted by Florese et al. whose results showed that the majority of scientific interest in Sport and Exercise Psychology is developed by Exercise and Sport Science programs rather than Psychology in Brazil universities, data that perhaps may drive us to have some reflection about the strength and power of the paradigm in which we are currently working. In this field, and for the first time in the scientific literature, Gavala-González et al. described the perception of the dual career in both professional and semi-professional under-23 canoeists. They encountered that athletes have specific problems that make it difficult to efficiently combine their athletic and academic careers. Certainly, both studies might serve as a precedent to intervene in the university to counteract the limitations of this field.

In a school context, one of the most intriguing debate is around the most suitable pedagogical model for a successful in physical education (Casey and MacPhail, 2018; González-Víllora et al., 2019). This issue is also addressed in this topic, specifically, Sierra-Díaz et al. tried in their systematic review to find implications of a model-based practice or instructional models on student's self-determined motivation and basic psychological needs. They concluded that model-based practice is an ideal pedagogical framework to produce significant increases in sport competence and self-determined motivation. More specifically, Gil-Madrona et al. studied the effects of an intervention based on a personal and social responsibility model. In brief, the intervention was effective to generate positive changes in children's attitudes and social skills. Lastly, Malkin et al. corroborate the importance of the physical activity to interfere in psychological factors, such as hardiness. These researchers found higher levels of hardiness in those students who engaged regularly in sports activities in comparison with those who did not. These studies enrich our understanding of physical activity and education binomial and offer new insights for developing interventions in physical education classes.

Collectively, this Research Topic has embraced a large spectrum of scientific concerns. Mostly, the publications of this Research Topic cover how psychological factors are influenced by physical activity, exercise and/or sport through a compendium of cross-sectional and systematic reviews studies. These works contributed toward the understanding of the impact of human movement on the physiological well-being, and also may show how the different fields placed under the umbrella of Sport Sciences interacted among them to produce and disseminate new knowledge both in theoretical and applied ways.

## Author Contributions

AM-M wrote the initial draft. AM-M, PP, PA, and AG-M review critically the final version of the manuscript.

## Conflict of Interest

The authors declare that the research was conducted in the absence of any commercial or financial relationships that could be construed as a potential conflict of interest.
